# Vigilanzminderung mit Hemiparese nach ZVK-Entfernung: eine seltene Komplikation

**DOI:** 10.1007/s00108-025-01936-y

**Published:** 2025-06-28

**Authors:** Josef Mohammad, Niklas Görnitz, Willi Karaszewski, Ariane Thon-Markau, Oliver Müller, Christian von Heymann, Georg Brinkhaus, Rolf Otto Reiter, Carsten Büning

**Affiliations:** 1 Charité – Universitätsmedizin Berlin, Charitéplatz 1, Berlin, Deutschland; 2 Abteilung für Innere Medizin, Krankenhaus Waldfriede, Berlin, Deutschland; 3 Klinik für Anästhesie, Intensivmedizin, Notfallmedizin und Schmerztherapie – Zentrum für hyperbare Sauerstofftherapie und Tauchmedizin, Vivantes Klinikum im Friedrichshain, Berlin, Deutschland; 4 Klinik für Radiologie, Charité – Universitätsmedizin Berlin, Hindenburgdamm 30, 12203 Berlin, Deutschland

Im Folgenden wird der seltene Fall einer ausgeprägten Luftembolie beschrieben, die nach der Entfernung eines zentralvenösen Katheters (ZVK) über das venöse System in die intrakraniellen venösen Gefäße gelangte und unmittelbar zu einer klinischen Symptomatik mit Vigilanzminderung und Hemiparese führte.

## Anamnese

Eine 71-jährige Patientin stellte sich mit Meläna, intermittierender Hämatochezie, Kältegefühl und schwerer Anämie (Hb 5,2 g/dl) und Kreislaufinstabilität in der Notaufnahme vor. Sie wurde unter dem Verdacht einer gastrointestinalen Blutung umgehend auf die Intensivstation verlegt. Aufgrund der hämodynamischen Instabilität unter dem Verdacht einer fulminanten gastrointestinalen Blutung und erwarteter Katecholamintherapie wurde ein High-flow-ZVK in die V. jugularis dextra gelegt. Nach Transfusion von Erythrozytenkonzentraten und FFP stabilisierte sich die Patientin. Eine anschließende Endoskopie identifizierte ein Ulcus duodeni Forrest 1b als Blutungsquelle. Nach erfolgreicher Stabilisierung und endoskopischer Therapie wurde die Patientin auf die Normalstation verlegt.

Im Verlauf der stationären Behandlung erfolgte dort die Entfernung des ZVK. Direkt im Anschluss verschlechterte sich der Zustand der Patientin rapide. Die Patientin entwickelte eine Vigilanzminderung, eine ausgeprägte Zyanose und zudem eine Hemiparese. Die plötzlich aufgetretene Symptomatik, klinisch am ehesten apoplektiform, veranlasste die sofortige Verständigung des Reanimationsteams.

## Untersuchung

Bei Eintreffen des Reanimationsteams war die Patientin bewusstlos mit einem Glasgow-Coma-Scale(GCS)-Wert von 8 Punkten, tachykard bei normotensivem Blutdruck und respiratorisch insuffizient (S_p_O_2_ 60 % trotz 8 l O_2_/min). Neurologisch fiel eine Blickdeviation nach rechts bei isokoren, mittelweiten Pupillen mit erhaltener Lichtreaktion auf.

## Diagnostik

Die differenzialdiagnostischen Überlegungen zielten zunächst auf einen akuten ischämischen Schlaganfall, da Blickdeviation, Hemiparese und Vigilanzminderung der Patientin mit einer supratentoriellen linkshemisphärischen Läsion vereinbar waren. Gleichzeitig wurde eine intrazerebrale Blutung in die Abwägung einbezogen, obwohl keine Antikoagulation oder relevante vaskuläre Vorgeschichte vorlag.

Als potenzielle iatrogene Komplikationen der ZVK-Entfernung wurden neben einer Luftembolie auch Gefäßwandverletzungen mit Thrombusdislokation, die Entwicklung eines Spannungspneumothorax oder einer Perikardtamponade diskutiert. Ein Hirnödem mit Einklemmungssymptomatik wurde aufgrund des Fehlens von Pupillenstörungen als Differenzialdiagnose weniger wahrscheinlich eingestuft.

**Abb. 1:**
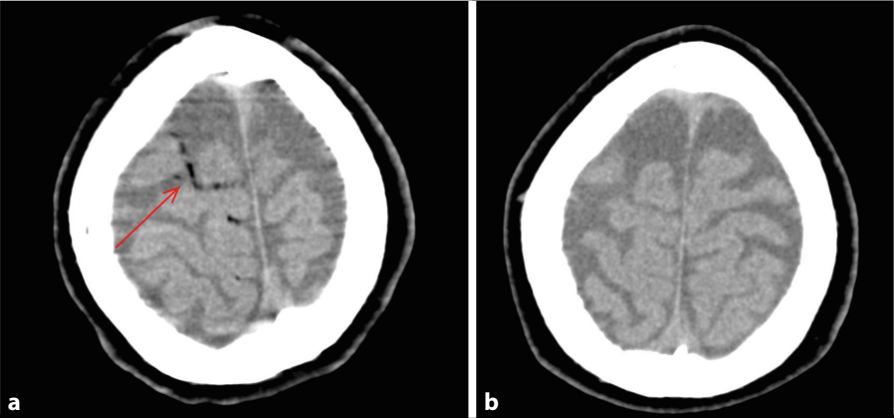
**a** Axiale kraniale CT-Aufnahme unmittelbar nach dem Ereignis. Darstellung von hypodensen, luftäquivalenten Strukturen in den oberflächlichen kortikalen Hirnvenen (siehe *roter Pfeil*), vereinbar mit einer zerebralen Luftembolie. **b** Kontroll-CT nach 17 h. Vollständige Regression der zuvor nachgewiesenen intrazerebralen Lufteinschlüsse, korrelierend mit der klinischen Besserung

Zum Ausschluss metabolischer, entzündlicher oder anderer neurologischer Genesen diente eine umgehende computertomographische Bildgebung (CT) des Kopfs mit Angiographie und Thorax-CT, in der sich luftsuspekte Läsionen im rechten Sinus cavernosus, hochfrontal und in der V. paravertebralis zeigten (Abb. 1a). Eine Lungenembolie wurde ausgeschlossen. Die Kontrastechokardiographie ergab weder intrakardiale Luftembolien noch einen Vorhofshunt, was die Diagnose einer retrograden zerebralen Luftembolie unterstützte.

## Therapie und Verlauf

Das Reanimationsteam begann eine manuelle Beatmung mit einem Beatmungsbeutel (Ambu-Beutel) und einem Sauerstofffluss (F_i_O_2_ 100 %) von 15 Litern pro Minute und Trendelenburg-Lagerung (−20°).

Diese Maßnahmen führten zu einem raschen Anstieg der Sauerstoffsättigung und zudem zu klinischer Verbesserung. Auf der Intensivstation zeigte die Patientin eine Spontanatmung bei stabiler Kreislaufsituation und Wachheit, jedoch mit persistierender linksseitiger Hemiplegie und ipsilateralem Neglect. Eine Therapieeskalation mittels hyperbarer Sauerstofftherapie stand aufgrund fehlender klinischer Kapazitäten nicht zur Verfügung.

Am Folgetag bildeten sich die neurologischen Symptome vollständig zurück, korrelierend mit der Regredienz der Luftembolie im Kontroll-CT (Abb. 1b). Bis auf eine retrograde Amnesie für den Vortag zeigte die mobilisierte Patientin keine neurologischen Defizite mehr.

## Diskussion

Luftembolien sind eine seltene, aber potenziell lebensbedrohliche klinische Komplikation. Neben dem Management von ZVK treten sie in der Geburtshilfe, perioperativ bei neurochirurgischen und kardiochirurgischen Eingriffen sowie bei endoskopischen Eingriffen mit Luftinsufflation auf [5].

Mit der zunehmenden Verwendung von zentralvenösen Zugängen im medizinischen Alltag steigt das Risiko dieser lebensbedrohlichen Komplikation. Insbesondere die Entfernung eines ZVK birgt ein hohes Risiko mit sich, mit einer Inzidenz von einer klinisch relevanten Luftembolie pro 800 Entfernungen [8].

Unser Fallbericht zeigt, wie schnell sich klinische Symptome entwickeln können und wie eine sofort eingeleitete Therapie zu einer vollständigen Remission der Symptome führte.

## Wie kommt es zur Luftaspiration?

Der Mechanismus der Luftembolie beruht auf der Aspiration von Luft durch eine offene Verbindung zwischen Punktionsstelle und V. cava superior. Ein physiologisch niedriger zentralvenöser Druck, der je nach Körperlage und Atemphase unter den atmosphärischen Druck absinken kann, begünstigt den Eintritt von Luft in das venöse Gefäßsystem. Risikofaktoren wie größere Katheter und längere Verweildauer erhöhen das Risiko zusätzlich. Fibrinablagerungen um den Katheter begünstigen die Bildung stabilerer Verbindungen und erleichtern so den Lufteintritt [9].

## Welche Wege der Embolie gibt es und wie treten diese in Erscheinung?

Im Gefäßsystem kann sich die Luftembolie unterschiedlich ausbreiten und verschiedene Organsysteme betreffen. Die Flussrichtung eines Luftembolus wird im Wesentlichen durch das Luftvolumen, den Gefäßdurchmesser und die hämodynamischen Verhältnisse bestimmt. In den meisten Fällen folgt der Luftembolus dem venösen Blutstrom (antegrad) zum rechten Herzen. Der retrograde Aufstieg von Luftblasen wird durch größere Luftvolumina (< 200 ml), weitlumige Venen oder reduziertes Herzzeitvolumen begünstigt. Der hydrostatische Druckunterschied beim Sitzen begünstigt ebenfalls den retrograden Aufstieg in den zerebralen Venensinus.

Venöse Embolien führen zu mechanischen Abflussbehinderungen, die sich klinisch oft in milden, reversiblen Symptomen äußern. Bei antegradem Transport einer Luftembolie zum rechten Herzen kann es jedoch je nach Volumen und Geschwindigkeit der Luftaspiration zu einer Obstruktion des Ausflusstrakts und damit zu einer Einschränkung der Auswurfleistung (Herzzeitvolumen) kommen. Auskultatorisch kann sich die mechanische Obstruktion als „Mühlradgeräusch“ über dem Herzen darstellen [11]. Zur Mobilisierung der Luftembolie aus dem rechten Ventrikel eignet sich hier das Durant-Manöver, bei dem in Linksseitenlage mit Trendelenburg-Lagerung eine Verlagerung der Luftembolie in den rechten Vorhof und damit eine Beseitigung der Obstruktion des rechtsventrikulären Ausflusstrakts erfolgt [8].

Gelangen Luftblasen mit dem Blutstrom in die Lunge, kommt es zur Okklusion arteriovenöser Kapillaren, wodurch ein weiteres Eindringen in den systemischen Kreislauf zunächst verhindert wird. Klinisch relevante Effekte treten bei Luftvolumina von ca. 100 ml auf, während Volumina ab 200 ml potenziell tödlich sind [8]. Die resultierende Dysbalance zwischen Ventilation und Perfusion führt zu einem Anstieg des pulmonalarteriellen Widerstands und damit zu einer akuten Rechtsherzbelastung. Diese manifestiert sich in einer Abnahme des rechtsventrikulären Schlagvolumens und einer Abnahme der linksventrikulären Vorlast und kann schließlich zu einer hämodynamischen Instabilität führen [11].

Der erhöhte Druck im rechten Herzen kann auch eine Wiedereröffnung des Foramen ovale begünstigen und damit einen intrakardialen Rechts-links-Shunt ermöglichen. Auch intrapulmonale Shunts können auftreten. Bei beiden Shuntformen besteht die Gefahr des Übertritts venöser Gasembolien in das arterielle System mit potenziell schwerwiegenderen Folgen [11].

Arterielle Embolien lösen durch direkte Okklusion endständiger Gefäße Ischämien aus, die sich in schwereren Verläufen manifestieren.

Neben der Reduktion der distalen Gewebeperfusion löst der Embolus eine komplexe Kaskade immunologischer und inflammatorischer Reaktionen aus. Dazu gehören die Aktivierung von Lymphozyten sowie die Freisetzung von Zytokinen und vasokonstriktorischen Mediatoren. Der Luftembolus wirkt als Fremdkörper, was zur Aktivierung des Komplementsystems und zu thromboinflammatorischen Prozessen führt. Fibrinablagerungen um den Embolus führen zu einer mechanischen Reizung des arteriellen Endothels, was intrakraniell zu einer Störung der Blut-Hirn-Schranke und damit zu vasogenen Ödemen und neuronalen Schäden über das ursprüngliche Obstruktionsgebiet hinaus führt [11]. Je nach betroffenem Areal kommt es zu fokalen neurologischen Ausfällen wie Hemiparesen und Hemianopsien, kognitiven Störungen und Bewusstseinsveränderungen. Auch Pupillenanomalien und Krampfanfälle können auftreten. Bei Beteiligung des Hirnstamms kann die kardiorespiratorische Regulation beeinträchtigt sein [11].

## Wie erfolgt die Diagnostik der Luftembolie?

In der vorliegenden Fallbeschreibung zeigte die CT-Untersuchung ausschließlich luftsuspekte Läsionen im intrakraniellen Venensystem, während pulmonale oder systemisch-arterielle Luftembolien nicht nachweisbar waren. Intrakranielle venöse Luftembolien sind typischerweise mit milden Symptomen assoziiert [3]. Bei ausgedehnter Obstruktion könnten diese jedoch durch einen venösen Abflussstau zu Atemdepression und Vigilanzminderung führen. Zusätzlich könnten thromboinflammatorische Prozesse, ausgelöst durch eine Endothelschädigung, die diffuse Hirnfunktionsstörung verstärkt haben. Venöse Infarkte wurden durch unauffällige Verlaufskontrollen ausgeschlossen.

Eine paradoxe Embolie über ein offenes Foramen ovale wurde erwogen, jedoch sprachen die Luftansammlungen im Sinus cavernosus und der Ausschluss eines Shunts in der Kontrastechokardiographie dagegen. Die diagnostische Sensitivität der CT ist bei kleinen Luftvolumina (< 1 ml) eingeschränkt, zumal diese innerhalb weniger Minuten resorbiert werden können [3]. Transiente systemisch-arterielle Luftembolien könnten daher ebenfalls eine mögliche Ursache der Atemdepression darstellen, während persistierende intrakranielle Luftblasen den venösen Abfluss beeinträchtigten.

Die rasche Regredienz der Vigilanzminderung ist untypisch für eine isolierte venöse Luftembolie und deutet auf ein multifaktorielles Zusammenspiel von mechanischer Obstruktion, inflammatorischen Prozessen und gegebenenfalls transienten arteriellen Embolien hin.

Für eine erweiterte Diagnostik von Luftembolien stehen verschiedene Verfahren zur Verfügung (siehe auch Tab. 1). Im chirurgischen Setting kann eine pulmonal gelegene Luftembolie zu einem Abfall des endtidalen CO_2_-Werts in der Kapnographie führen. Eine weitere Methode ist der Nachweis von Stickstoff in der Ausatemluft bei Beatmung mit 100 % Sauerstoff [5]. Ventilations-Perfusions-SPECT/CT-Untersuchungen sind in der Lage, Perfusionsdefekte bei erhaltener Ventilation mit sehr hoher Sensitivität darzustellen [4].

**Tab. 1:** Übersicht der diagnostischen Methoden zur Detektion von Luftembolien

Methode	Vorteile	Nachteile	Indikationen	Mindestgröße Luftvolumen
*Transösophageale Echokardiographie (TEE)*	Höchste Sensitivität, erkennt kleine Luftmengen, Goldstandard für PFO [5, 7, 10]	Invasiv, erfordert Expertise [7, 10]	Nachweis von Luftembolien im Herzen und in großen Gefäßen [5]	0,02 ml/kg [7, 10]
*Präkordialer Doppler*	Hohe Sensitivität, nichtinvasiv, schnelle Anwendung [7, 10]	Störanfällig (z. B. durch OP-Umgebung), eingeschränkte Nutzung bei Adipositas [7, 10]	Detektion von venösen Luftembolien intraoperativ [7, 10]	0,05 ml/kg [7, 10]
*Transkranieller Doppler (TCD)*	Sensitiv für zerebrale Luftembolien, ermöglicht Nachweis von Rechts-links-Shunts [7]	Erfordert Erfahrung, nicht überall verfügbar [7]	Erkennung von Luftembolien im zerebralen Kreislauf [7]	0,05 ml/kg [1, 7]
*Pulmonalarterienkatheter (PA-Katheter)*	Erlaubt Messung hämodynamischer Effekte, sensitiv für größere Luftmengen [7, 10]	Invasiv, Risiko durch Katheterlage [7, 10]	Hämodynamische Überwachung bei Hochrisikopatienten [7, 10]	0,25 ml/kg [7, 10]
*Endtidale CO*_*2*_*-Messung (ETCO*_*2*_*)*	Schnell verfügbar, nichtinvasiv [7, 10]	Unspezifisch für Luftembolien, kann durch Perfusionsprobleme beeinflusst sein [7, 10]	Indirekter Hinweis auf Luftembolien durch plötzlichen Abfall des CO_2_ [7, 10]	0,5 ml/kg [7, 10]
*Endtidale N*_*2*_*-Messung (ETN*_*2*_*)*	Relativ spezifisch für Luftembolien [7]	Nicht überall verfügbar, beeinflusst durch Beatmungseinstellungen [7]	Nachweis von Luft bei Beatmung mit 100 % O_2_ [5]	0,5 ml/kg [7]
*CT-Angiographie*	Gute Spezifität für venöse Luftembolien [10]	Begrenzte Sensitivität für kleine Luftmengen (< 1 ml), kann transiente Embolien übersehen [3]	Diagnostik von venösen und arteriellen Luftembolien [10]	0,5 ml/kg [10]
*EKG*	Einfach verfügbar, erkennt Luftembolien in Koronararterien durch Dysrhythmien [5]	Unspezifisch, kann andere kardiale Ereignisse nicht sicher ausschließen [5]	Nachweis von Luftembolien mit kardialer Beteiligung [5]	1,25 ml/kg [10]
*SPECT/CT*	Hohe Sensitivität für Perfusionsdefekte [4]	Nicht immer verfügbar, zeitaufwendig [4]	Unterscheidung von perfundierten und nichtperfundierten Lungenarealen [4]	–
*Direkte Visualisierung (chirurgisch/endoskopisch)*	Direkter Nachweis möglich [10]	Keine quantitative Aussage, hohe Abhängigkeit von OP-Bedingungen [10]	Identifikation von Luft in Operationsgebieten [10]	–
*Ösophageales Stethoskop/präkordiales Stethoskop*	Günstig und einfach verfügbar [7, 10]	Niedrige Sensitivität, nur bei großen Luftembolien effektiv [7, 10]	Detektion von ausgeprägten Luftembolien durch „Mühlradgeräusch“ [7, 10]	1,5 ml/kg [10]

Bei Lokalisation der Luftembolie in den Koronararterien kann es zu einer Dysrhythmie und damit zu infarkttypischen EKG-Veränderungen kommen [5]. Eine Luftembolie innerhalb der Herzkammern kann zu dem oben beschriebenen auskultatorisch nachweisbaren Mühlradgeräusch führen. Die sensitivste Methode zur Darstellung von Luftembolie im Herzen ist die transösophageale Echokardiographie, die bereits Luftvolumina ab 0,01–0,19 ml/kg darstellen kann [5].

Trotz der hohen Spezifität der CT-Angiographie für venöse Luftmikrobläschen (ab 0,5 ml/kg) in thorakalen Gefäßen bleiben Mikroembolien in arteriellen Gefäßen oder transient resorbierte Luftmengen häufig unentdeckt [3]. Dies unterstreicht die Notwendigkeit einer symptomorientierten Therapieentscheidung auch bei negativem CT-Befund.

## Wie erfolgt die Behandlung der ZVK-assoziierten Luftembolie?

Bei ZVK-assoziierten Luftembolien besteht die primäre Interventionsmaßnahme im sofortigen luftdichten Verschluss der Punktionsstelle mit geeignetem Kompressions- oder Verbandmaterial. Unmittelbar daran schließt sich die Applikation von Sauerstoff mit hoher Flussrate an. Die Erhöhung des Sauerstoffpartialdrucks führt neben einer verbesserten Gewebeoxygenierung zu einer beschleunigten Diffusion von Stickstoff aus dem Embolus und damit zu einer Reduktion des Embolusvolumens [12].

Bei venöser intrakranieller Lage unterstützt eine Kopftieflagerung den Auftrieb der Luftblase. Bei hirnarterieller Embolie reicht die Kopftieflagerung jedoch nicht aus, um dem Blutfluss entgegenzuwirken. Da die Kopftieflage ein Hirnödem verstärken kann, sollten Patienten mit arterieller intrakranieller Gasembolie flach gelagert werden. Die hyperbare Sauerstofftherapie (HBOT) sollte bei nachgewiesener symptomatischer Luftembolie die Therapie der Wahl sein. Sie erhöht den Sauerstoffpartialdruck auf supraphysiologische Werte bis ~2000 mm Hg, beschleunigt die Stickstoffdiffusion und die Reduktion des Embolusvolumens. Die Indikation zur HBOT sollte umgehend in Absprache mit dem nächstgelegenen Zentrum geprüft werden [6]. Auch nach 24 h bleibt der therapeutische Nutzen der HBOT aufgrund der immunmodulatorischen Effekte relevant.

Bei der venösen intrakraniellen Lage eines Luftembolus ist die Erhöhung der Sauerstofffraktion auf 100 % die primäre Therapie. Tritt jedoch keine rasche Besserung der neurologischen Symptome ein, sollte umgehend ein HBOT-Zentrum kontaktiert werden. In Anlehnung an die Leitlinie für Tauchunfälle sollte bei persistierenden leichten neurologischen Symptomen nach spätestens 30 min eine Reevaluation zur Therapieeskalation erfolgen, bei schweren Symptomen sofort [2].

## Wie können Luftembolien verhindert werden?

Um das Risiko dieser schwerwiegenden Komplikation nach ZVK-Entfernung zu minimieren, sollte der zentralvenöse Druck durch flache bis kopftiefe Lagerung erhöht werden. Während der ZVK-Entfernung sollte der Patient langsam ausatmen. Bei Patienten mit Hustenreiz ist die Gabe eines Antitussivums vor der ZVK-Entfernung indiziert. Nach der ZVK-Entfernung ist die Austrittsstelle für mindestens 5 bis 10 min mit einem luftdichten Verband zu verschließen und ggf. zu komprimieren. Für mindestens weitere 30 min sollte der Patient flach gelagert werden [8].

Eine effektive Prävention von Luftembolien setzt bereits bei der kritischen Indikationsstellung zur ZVK-Anlage an. Die Implementierung einer klinikweiten standardisierten Handlungsanweisung (SOP) gewährleistet zudem ein schnelles und zielgerichtetes Vorgehen bei der Prophylaxe und Therapie der Luftembolie.

## Fazit für die Praxis

- Schulung des Personals: Das Personal sollte über die Risiken einer zerebralen Luftembolie als Komplikation der ZVK-Entfernung, insbesondere über die verminderte Vigilanz und kardiovaskuläre Symptome als Frühwarnzeichen, geschult werden.
- Sofortintervention: Bei Verdacht auf Luftembolie nach ZVK-Entfernung sofortige suffiziente Kompression der Punktionsstelle, Trendelenburg-Lagerung bei Verdacht auf venöse Lage der Luftembolie und hoch dosierte Sauerstofftherapie.
- Bei symptomatischem Luftembolus sollte niedrigschwellig eine Kontaktaufnahme mit einem HBOT-Zentrum zur Abklärung der Therapieoptionen erfolgen.
